# Status and Trend of Regal Fritillary (*Speyeria idalia*) (Lepidoptera: Nymphalidae) in the 4th of July Butterfly Count Program in 1977–2014

**DOI:** 10.1155/2016/2572056

**Published:** 2016-04-28

**Authors:** Scott R. Swengel, Ann B. Swengel

**Affiliations:** 909 Birch Street, Baraboo, WI 53913, USA

## Abstract

Regal Fritillary (*Speyeria idalia*) primarily inhabits prairie, a native grassland of central North America, and occurs rarely in nonprairie grasslands further east. This butterfly has experienced widespread decline and marked range contraction. We analyze Regal Fritillary incidence and abundance during 1977–2014 in 4th of July Butterfly Counts, an annual census of butterflies in North America. Volunteers count within the same 24 km diameter circle each year. Only 6% of counts in range reported a Regal, while 18% of counts in core range in the Midwest and Great Plains did. 99.9% of Regal individuals occurred in core range. Only four circles east of core range reported this species, and only during the first half of the study period. All individuals reported west of its main range occurred in two circles in Colorado in the second half of the study. The number of counts per year and survey effort per count increased during the study. During 1991–2014, >31 counts occurred per year in core Regal range, compared to 0–23 during 1975–1990. During 1991–2014, all measures of Regal presence and abundance declined, most significantly. These results agree with other sources that Regal Fritillary has contracted its range and declined in abundance.

## 1. Introduction

Patterned after Christmas Bird Counts, 4th of July Butterfly Counts (4JCs) began in 1975 as an annual, international census of butterflies in North America [1]. Volunteers initiating a count establish a 15-mile (24 km) diameter count circle, which remains the same each year the count is conducted, although actual count sites within the circle may vary from year to year. On a single date, participants record the number of each species of butterfly (“true butterflies” or “scudders,” superfamily Papilionoidea, and skippers, superfamily Hesperioidea) seen, weather, number of observers in how many field parties, and how much time each party spent in the field (called “party-hours”). 4JCs are held once per year usually during June and July, but some count circles have traditional count dates outside that period that have remained relatively the same over time. Results are published annually (the Appendix).

Swengel [1] reviewed methods for standardizing data from 4JCs to support statistical analysis of incidence and abundance patterns. Although this program was intended to be recreational, midwestern USA count compilers have treated it seriously as a way to study, conserve, and educate about butterflies [1]. 4JCs are relatively informal and typically cover a better than average sample of the landscape because observers want to find more species and more individuals. There are, however, large numbers of urban 4JCs that offset to some degree the positive bias in habitats chosen by exurban observers. Despite count results being relatively informal, they have been cross-validated to other data sources [2–6]. As a result, count results are scientifically useful for a variety of biogeographical topics, including Monarch abundance [2, 5–8], large-scale spatial synchrony of populations [7], distribution and abundance of butterfly mimicry complexes [8], and large-scale biogeography and habitats of butterflies [2, 4, 9, 10].

Several survey methods can be used to study the status and abundance of butterflies. In standardized transects (“Pollard Walks”), butterflies are counted within a fixed width (e.g. 2.5 meters ahead and to each side of the line walked) weekly in spring and summer along a standardized route so that results are comparable over time and can be used to estimate relative changes in abundance over time [11]. This survey method is used in many butterfly monitoring programs, such as in Great Britain and Ohio, USA (e.g. [8, 11]). In checklist or meandering surveys, observers search any place in a site likely to have butterflies, on any pathway for any length of time, with a goal of finding as many butterfly species or individuals as possible [12]. Checklist surveys are more efficient than Pollard Walks for compiling a more complete species list at a site. 4JC participants use variants of these two methods to count a large number of both species and individuals within a circle in one day ([1] and personal observation). Some butterfly monitoring schemes also allow counters the freedom to count using different methods that they then use consistently at a site over time, for example, Illinois, USA [8].

Pollard Walks can be changed to unlimited width counting strips along set routes so that all butterflies seen are included from the count. The surveys may also be done less frequently, for example, targeting the seasonal timing of key species or greatest species richness [13, 14]. These modified methods result in butterfly abundance data that are significantly positively correlated with the results from other survey methods and with the results from the mixed counting techniques used on 4JCs [2, 6, 15].

**Figure 1:**
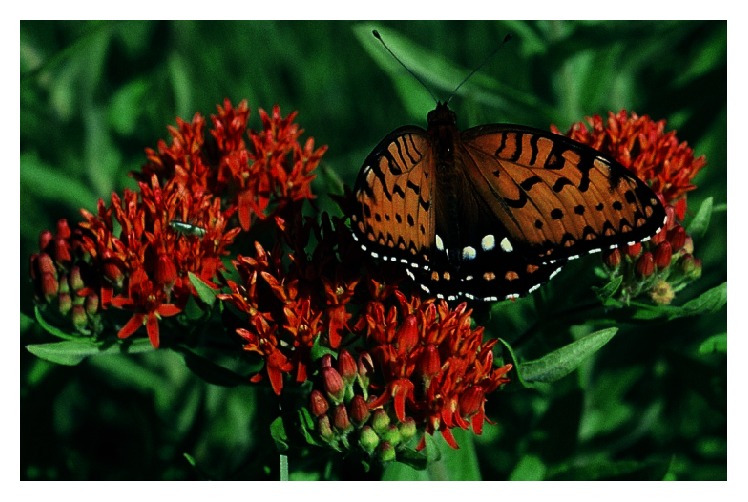
Dorsal view of a Regal Fritillary nectaring on Butterfly Milkweed (*Asclepias tuberosa*) in Missouri, USA, showing the submarginal row of orange spots on the hindwing in males (white in females). Photo by Ann B. Swengel.

**Figure 2:**
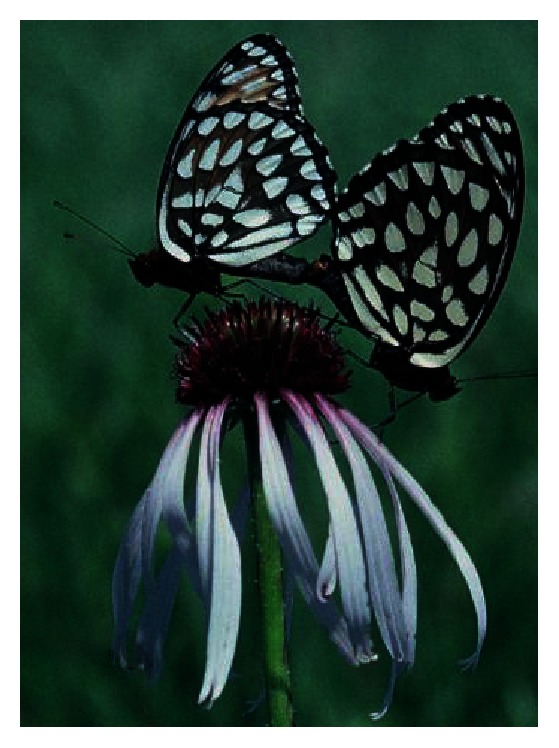
Ventral view of Regal Fritillaries mating while perched on Pale Purple Coneflower (*Echinacea pallida*) in Missouri, USA, with female on right (larger and darker in the forewing tip than the male). Photo by Ann B. Swengel.

Because most analyses of 4JC data are based on large numbers of counts and years, the sampling effort is usually very large. This dataset is the only way to obtain continent-wide butterfly data compiled the same way and with measures of survey effort. Wells and Tonkyn [16] took advantage of 4JCs' large geographical coverage to demonstrate range contraction in the specialized Diana Fritillary (*Speyeria diana*), a congener of the Regal Fritillary (*S. idalia*). This recent range contraction was corroborated by independent data sources from the last two centuries. The Regal Fritillary (Figures 1 and 2) is also particularly suitable for analysis in 4JC because it is a large and strikingly identifiable butterfly that has a long “flight period” (time of year when in the adult life stage) broadly spanning the summer timing of most counts [17, 18].

Regal Fritillary primarily inhabits prairie, a grassland habitat of native floristic composition in central North America. Outside the prairie region it occurs in a localized manner in damp meadows and upland pastures, not necessarily of native vegetation types [17, 18]. Because of the vast destruction of prairie in the past two centuries mostly for conversion to agriculture, the Regal Fritillary has experienced widespread decline and marked range contraction [19–27]. As a result of this conservation concern, much survey work has been conducted to assess this species' status and trend [28–35].

In this paper, we analyze trend over time in annual incidence and abundance of Regal Fritillary in 4JC data. The results of this analysis should be useful for assessing long-term status and trend of the Regal Fritillary throughout its range. This analysis is timely because the Regal Fritillary's status and trend are currently being reviewed by the United Stated Fish and Wildlife Service [36].

## 2. Methodology

### 2.1. Data

We searched all 4JC reports of 1975–2014 for Regal Fritillary data (see the Appendix for citations of all annual published count reports). We confirmed that the number of counts reporting any Regals we found was the same number of counts identified in summary statistics in each 4JC report tabulating number of counts reporting any Regals. We then added Regal data for counts published in the Late Counts section of each report, where counts from prior year(s) are published. At least once, a year's national high for total Regal individuals on a single count was increased by a count reported late. Starting in 2007, the count program expanded from one count period per year to three (spring, midsummer, and late summer). For circles reporting more than once per year, we selected the count (summer or late summer) more consistent in timing with previous years of that count (most always occurred in the midsummer period).

**Figure 3:**
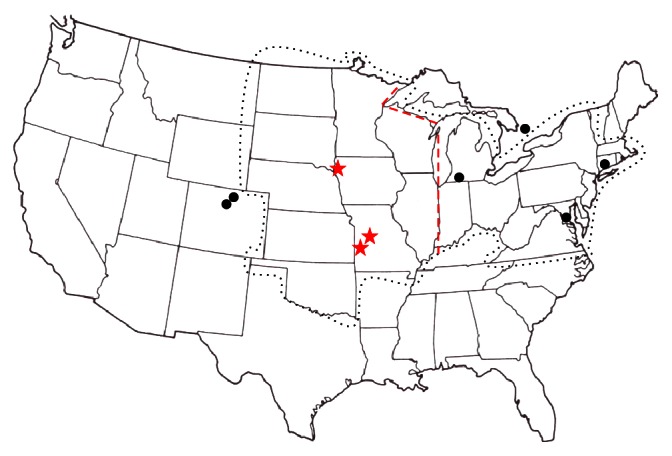
Map showing states containing main Regal Fritillary range (encircled by black dotted line), core range (west of red dashed line), all count circles reporting Regal Fritillary outside core range (black circles), and the three count circles with highest Regal abundance (red stars).

Based on reference books [17, 18], we defined the main range of Regal Fritillary (Figure 3) as states south of Canada; east of Colorado, Wyoming, and Montana; north of Texas, Arkansas, Kentucky, and North Carolina; and excluding Vermont, New Hampshire, and Maine. This excluded large areas of the count program in the USA, Canada, and Mexico that had virtually no chance of finding a Regal. We defined core range as the western half of the main range (Figure 3): Illinois, Iowa, Kansas, Missouri, Minnesota, Nebraska, North Dakota, Oklahoma, South Dakota, and Wisconsin.

We compiled statistics for each year on number of counts in all of North America, main Regal range, and core Regal range. We calculated the percent of counts reporting any Regals and total Regal individuals seen (all, main, and core range), peak Regal individual total on a single count, Regal individuals per party-hour on all counts reporting Regals, and number of counts reporting ≥100 and ≥500 individuals. We also databased summary statistics on all counts in core range each year to assess survey effort on counts over time in the region where most Regal Fritillaries were found. Analysis of this dataset, including Regal individuals per total party-hours in core range, could determine whether simply using the number of 4JCs on which Regals were eligible to be found was likely to be a valid comparison, or whether Regal abundance needed to be indexed to total party-hours of effort.

For each circle that ever reported a Regal, we databased every year of that count with these variables: circle name abbreviation, latitude, longitude, date, party-hours, and number of Regals reported (including zero). We calculated *N* years reported for these circles, first and last year reported, first and last year Regal reported, and percent of these counts reporting any Regals, total Regal individuals seen, and Regal individuals per party-hour.

### 2.2. Data Analyses

All analyses were done with ABstat 7.20 software (Anderson-Bell Corp., Parker, Colorado, 1994). All tests were two-tailed, with statistical significance set at *P* < 0.05. Since significant results occurred at a frequency well above that expected due to spurious Type I statistical error, the critical *P* value was not lowered further, as more Type II errors would be created than would Type I errors be eliminated. In 1975, no counts occurred in core Regal range, and, in 1976, reporting of numbers seen was inconsistent. Thus we did statistical testing for the period 1977–2014. We analyzed trend and rate of change of Regal Fritillary presence and abundance measures using Spearman rank correlation and linear regression. To compare presence and abundance measures, we used the Pearson product moment and Spearman rank correlations. For the parametric tests, we natural-log-transformed the dependent variables because they were not normally distributed. We used both parametric and nonparametric analyses to test for both linear and nonlinear patterns.

## 3. Results

**Figure 4:**
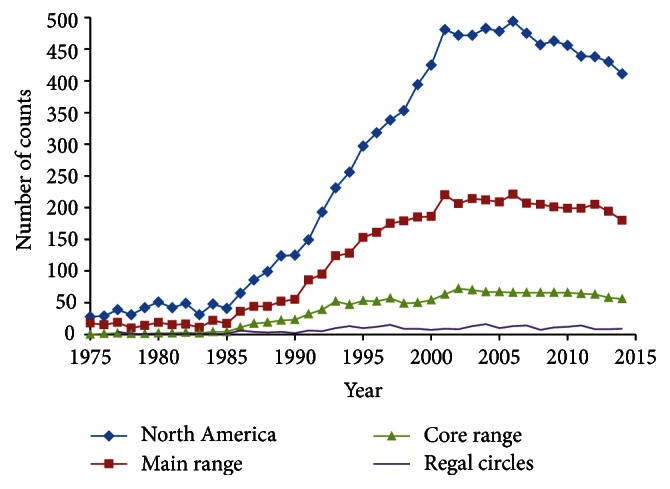
Number of counts per year in North America, main Regal range, core Regal range, and count circles ever reporting a Regal Fritillary.

The number of counts per year increased dramatically during the first 25 years of the program (Figure 4). The number of counts in main and especially core Regal range was quite low in the early decades of the program. Effort per count (number of observers and party-hours) also increased during the study period (Table 1), even in 2001–2014, after the rapid growth of the program ended.

Of all Regal individuals reported in the 4th of July count program, 99.9% occurred in the area defined as core range (Table 2). Of the 60 count circles ever reporting any Regals, 54 (90%) were in core range and 57 (95%) in main range. Only five Regal individuals occurred outside main range (Figure 3): four in eastern Colorado (west of main range) on three counts in two circles (Fort Collins in 1997-1998, Roosevelt National Forest in 2008) and one in southern Ontario (Orillia in 1994). In main range east of core range, Regal Fritillary only occurred in three circles (Figure 3): twice in St. Joseph County, Michigan (21 individuals 1977-1978), and one individual each in Woodbridge/Bethany, Connecticut (1977) and Prince George's County, Maryland (1993). In main range, only 6% of counts reported a Regal, while 18% of counts in core range did (Table 2). Seven of the 60 circles ever reporting a Regal had a count in only one year. Of the remaining 53 circles, 16 reported only one Regal individual once.

**Figure 5:**
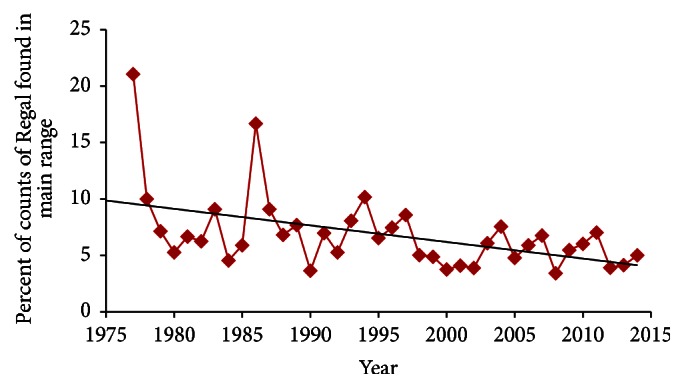
Percent of counts reporting Regal Fritillary each year in main Regal range, 1977–2014, with linear regression trend line. This declined significantly in both periods analyzed (Table 3).

**Figure 6:**
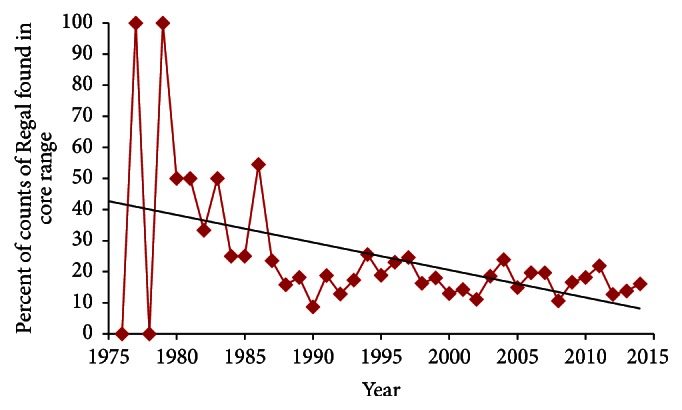
Percent of counts reporting Regal Fritillary each year in core Regal range, 1977–2014, with regression trend line. This decline was significant in 1977–2014 but not in 1991–2014 (Table 3).

**Figure 7:**
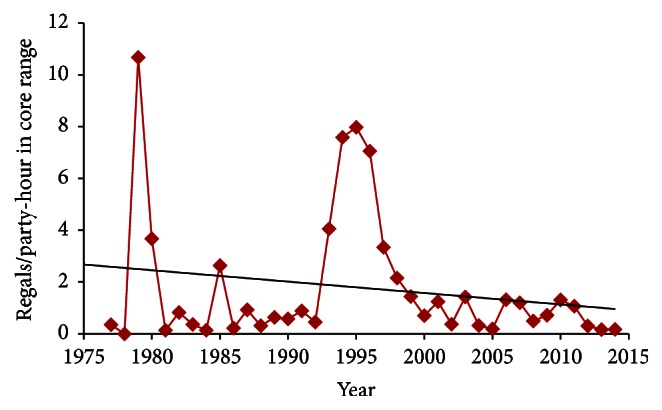
Total Regal Fritillary individuals per total party-hours reported in core range each year, with regression trend line. This decline was far from significant in 1977–2014 but was significant in 1991–2014 (Table 3).

**Figure 8:**
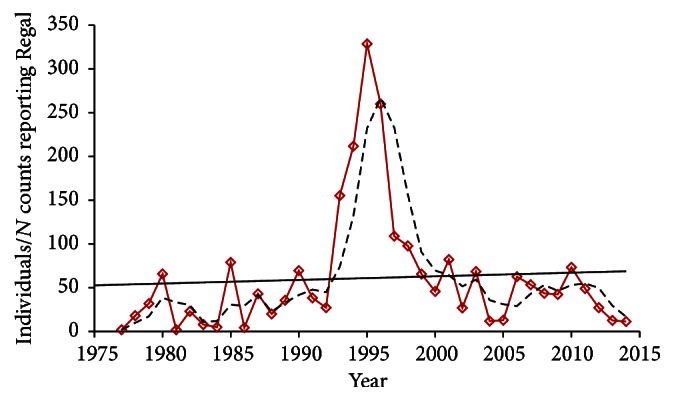
Total Regal individuals divided by number of counts reporting Regal present, 1977–2014, with regression trend line and three-year running average. The three counts in the highest order of magnitude abundance category (Table 4) were held in 1993–99 (but in no year were all three counts held) with one also reporting in 2002. This was weakly positive but far from significant in 1977–2014; the decline was significant in 1991–2014 (Table 3).

During 1991–2014, a minimum of 32 counts occurred per year in core Regal range, compared to 0–23 during 1975–1990 (Figure 4). During 1991–2014, all measures of Regal occurrence and abundance declined over time (Table 3), significantly so in most cases. Some of these measures decreased during the entire study period as well, a few of those significantly so (Table 3, Figures 5–8). However, a few measures per count increased, even significantly, when including the early years of the count program in analysis (Table 3, Figure 8). Those apparent increases primarily related to abundance. Disproportionately few count circles (11/60) achieved the two highest abundance orders of magnitude (Table 4). These 11 circles also disproportionately were held for fewer years and ceased reporting earlier than the other counts (Table 4). The three counts with the highest Regal abundance were most represented just after the midpoint of the study period (Table 4).

Regal presence correlated positively with abundance (Regal individuals per count) (Table 5). This pattern was relatively weak over the entire study period but extremely strong during 1991–2014 (*P* < 0.01 in all comparisons). During the entire study, the regression line indicated a 56% reduction in percent of counts with Regal presence in main range (Figure 5) and 80% reduction in core range (Figure 6). Likewise, the regression line indicated a 62% reduction in Regals per party-hour in core range (Figure 7). But Regal individuals per count reporting Regals that year showed a 27% increase during 1977–2014 (Figure 8). However, the three-year running average (Figure 8) indicated a large increase in the middle 1990s followed by a large decrease. When linear trend was calculated separately for the earlier (1977–1991) and later (1991–2014) periods, the regression line indicated a 174% increase earlier but a 94% decline later, or a net effect of 83% decline over the entire study period.

## 4. Discussion

The 4th of July counts provided the tremendous statistical advantage of time depth and geographic breadth for assessing Regal incidence and abundance. All measures of Regal trend were negative to some degree during 1991–2014 (Table 3, Figures 5–8). This justifies further investigation of the Regal's status and trend currently underway [36].

However, limitations of 4th of July count data were apparent in this analysis. The counts with the highest abundance of Regals were also the ones held the fewest years and discontinued sooner in the study period (Table 4, Figure 8). The three counts in the highest abundance category were held only in the middle of the study period (Figure 8). This appears to contribute to the disparity between all trends being negative to some degree during 1991–2014 while some were positive to any degree during the entire study period (Table 3). It is not possible to address how Regal trend would appear if more high-abundance circles had reported for more years of the count program. As it is, the great majority of circles reporting any Regals had had low Regal abundance. The turnover in which circles reported results each year precluded holding location constant to assess trend over time. Counts have been biased toward areas of higher human population density, rather than being evenly spread throughout the continent. When relatively fewer counts were held earlier in the study period, this resulted in few counts in core Regal range and habitat (Figure 4).

Nonetheless, even as number of counts and counting effort per count increased during the study period (Table 1, Figure 4), most measures of Regal presence and abundance decreased during the study period (Table 1, Figures 5–8). Since the likelihood of finding any Regals on a count should increase with increasing effort per count, the opportunity per count to find any Regal increased over time. But as the number of count circles increased during the study, it is possible that the new circles added over time were on average of slightly lower quality than the earlier circles formed when the entire landscape was available to select from. However, in the area of highest Regal abundance in the western tallgrass prairie zone, the vast majority of land remained unsurveyed by any 4JC circles. If there is a circle quality effect, this may be offset by the increase in observers and party-hours per count during the study period.

Abundance measures (Figures 7 and 8) may have been skewed earlier in this study due to a combination of low sample (Figure 3) and turnover (Tables 2 and 4) of circles. But later in this study (1991–2014), presence-absence measures (Figures 5 and 6) may have underestimated the decline in population size because the diminishing proportion of counts with Regal presence also had far fewer Regals per count over time. Regal presence-absence had a relatively strong negative trend over the whole study and an even stronger decline more recently (1991–2014) (Table 3). Abundance measures, however, had relatively neutral to slightly positive trends over the whole study but steeply negative trends during 1991–2014 (Table 3). In a transect survey dataset of prairie butterflies, patterns of presence-absence and abundance largely agreed but abundance was the more powerful statistical measure [37]. However, for a different prairie butterfly with a highly skewed distribution of population abundances, both types of measure were valuable and complementary in characterizing the species' status and trend [38]. In 4JCs, Regal Fritillary was similarly skewed in distribution of population abundances (Table 4).

In addition to the trend results, another indication of Regal decline is its range contraction westward. All individuals on counts east of core range (Figure 3) occurred during 1977–1994 even though the number of counts during 1995–2014 was much greater than during 1977–1994 (Figure 4). This decline in counts in the eastern range before 1995 is consistent with the earlier large decline and range contraction reported in the East [21, 28, 34] compared to its western range. Also notable is that >85% of Regal individuals ever found on counts east of core range were in St. Joseph County, Michigan, not far east of core range (Figure 3).

By contrast, all individuals reported west of main range occurred in Colorado in the second half of the study period (1997, 1998, and 2008). Thus, either range expansion or increase in abundance at range edge may have occurred there.

Although Regals declined more and earlier in the East than in core range both in this study and as documented by others (discussed above), there are indications of important declines in parts of core range. A 1995 survey was unable to find Regals in 41/52 (79%) of historic Regal sites checked in Iowa [24]. After that, Regal counts declined by about 75% on a constant set of Iowa sites between 1989–96 and 2004–07 [35]. Many historical Regal sites in Wisconsin appeared to be extirpated by 1999 [30] and most Regal populations known in preserves as of 1990 had declined by 2009 [35]. However, species-specific management produced some increases or long-term stability [32, 35, 39]. Powell et al. [33] reviewed Regal status in much of its core range and determined that the literature did not demonstrate a large recent decline additive to the approximately 99% outright decline in its prairie habitat from human development in the last two centuries. They found Regals in 80% of small prairies checked in northeastern Kansas in 2005. This is consistent with the relatively high abundance found in the 1990s in Nebraska and western Missouri [40, 41] (Figure 1). Very high Regal abundances in southwestern Missouri in 1992–99 [41] may have declined a decade or more later in some of the same sites [42]. The comparison is approximate because the two research teams used different method to survey and calculate abundance. But this comparison suggests a possible decline as rotational haying in the earlier study period was replaced with burning, combined with grazing in some parts of some sites, before the later study period. This likely Regal decline after preserve management changed from haying to fire is consistent with findings in a nearby Kansas study according to which Regals were far less abundant in burned than unburned sites [33].

## 5. Conclusion

Regal Fritillary data in 4JCs generally agreed with other sources in that Regal Fritillary is a localized butterfly that has contracted in range and declined within its existing range. Analysis of 4JCs contributes to status and trend assessment by providing geographic breadth and time depth. 4JCs are more suitable for statistical analysis than opportunistic citizen-science reporting programs because 4JCs report complete species lists and consistently measured survey effort [43]. But targeted and consistent formal surveying at key sites, as promoted by the North American Butterfly Monitoring Network (http://www.nab-net.org/), is also needed to document fully the status and trend of this butterfly.

## Figures and Tables

**Table 1:** Spearman rank correlations of measures of count effort with year (trend over time) in core Regal range.

	1977–2014			2001–2014		
	*N* counts	*R*	*P*	*N* counts	*R*	*P*
Number of observers per count	1509	+0.1589	<0.001	910	+0.0966	<0.01
Party-hours per count	1507^*∗*^	+0.1627	<0.001	910	+0.1077	<0.01
Party-hours per count in circles ever reporting a Regal	718	+0.113	<0.01	414	+0.0522	>0.10

^*∗*^2 counts were missing party-hours in the reports.

**Table 2:** Summary statistics on 4JC circles ever reporting a Regal Fritillary and all counts reported from those circles, by geographic scope.

	North America	Main range	Core range
*N* circles with Regal Fritillary reported	60	57	54
*N* counts reported from those circles	719	669	646
Total Regal individuals reported	21,783	21,778	21,755
Total party-hours in these circles	7237.59	6590.66	6064.66
*N* counts reporting Regal present	279	275	271
Median *N* years reported per circle	12	11	11.5
Median first year held	1993	1993	1993
Median last year held	2012	2012	2012
*N* counts from all circles in region	10,333	4751	1509

**Table 3:** Results of linear regressions and Spearman rank correlations of dependent variables versus year. NS = not significant.

	Linear regression standardized coefficient (*R*)	*P*	Spearman rank correlation coefficient (*R*)	*P*
1977–2014 (*N* = 38 years)				
Percent counts per year with Regal reported:				
North America	−0.358	0.0272	−0.303	<0.10
Main range	−0.514	0.0010	−0.482	<0.01
Core range	−0.022	0.8899	−0.509	<0.01
Percent counts per year in main range reporting:				
≥100 Regal individuals	+0.285	0.0827	+0.309	<0.10
≥500 Regal individuals	+0.008	0.7621	+0.088	NS
Regal individuals reported that year divided by:				
* N* counts that year in circles ever reporting Regal	+0.264	0.1094	+0.210	NS
* N* counts held in main range	+0.074	0.6580	+0.091	NS
Total party-hours on all counts in core range	−0.106	0.5281	−0.014	NS
Highest Regal total on a single count	+0.528	0.0007	+0.472	<0.01

1991–2014 (*N* = 24 years)				
Percent counts per year with Regal reported:				
North America	−0.561	0.0043	−0.527	<0.01
Main range	−0.405	0.0497	−0.383	<0.10
Core range	−0.244	0.2511	−0.255	NS
Percent counts per year in main range reporting:				
≥100 Regal individuals	−0.528	0.0080	−0.563	<0.01
≥500 Regal individuals	−0.677	0.0003	−0.671	<0.01
Regal individuals reported that year divided by:				
* N* counts that year in circles ever reporting Regal	−0.571	0.0036	−0.517	<0.01
* N* counts held in main range	−0.590	0.0024	−0.523	<0.01
Total party-hours on all counts in core range	−0.611	0.0015	−0.600	<0.01
Highest Regal total on a single count	−0.496	0.0136	−0.519	<0.05

**Table 4:** Number of circles in each category of Regal abundance (calculated as total Regal individuals reported per total party-hours), mean and median year that a count was last reported from those circles, and that Regal Fritillary was last reported in those circles. Spearman rank correlations of total Regal individuals per total party-hours per circle with last year reported (*r* = −0.41920, *P* < 0.01) and with last year Regal reported (*r* = +0.03631, *P* > 0.10) and with number of years reported (*r* = −0.50137, *P* < 0.01), *N* = 60 for all.

Total Regals per party-hours	*N*	Last year reported		Last year Regal found		Median number of years reported
	Circles	Mean	Median	Mean	Median	
124–160	3	1997.3	1997	1997.3	1997	4
11–50	8	1999.9	1996.5	1999.9	1996.5	3
1–8	12	2007.4	2007.3	2012	2011.5	9
0.1–0.8	13	2001.2	2000.4	2004	2004	10
0.01–0.09	11	2008.6	2003.4	2014	2007	17
0.001–0.009	13	2011.9	2001.1	2014	2003	18

**Table 5:** Results of Pearson product moment and Spearman rank correlations of a Regal Fritillary abundance variable to presence-absence (percent) variables. NS = not significant.

	Pearson		Spearman rank	
	*R*	*P*	*R*	*P*
1977–2014 (*N* = 38 years)				
Regal individuals reported that year divided by *N* counts in main range with:				
Percent counts with any Regal reported that year				
North America	+0.20196	0.2240	+0.34446	<0.05
Main range	+0.06216	0.7108	+0.16635	NS
Total Regals per party-hour in core range with:				
Percent counts with any Regal reported that year in core range	+0.31101	0.0574	+0.28009	<0.10

1991–2014 (*N* = 24 years)				
Regal individuals reported that year divided by *N* counts in main range with:				
Percent counts with any Regal reported that year				
North America	+0.73457	0.0000	+0.70102	0.0000
Main range	+0.65802	0.0005	+0.59478	<0.01
Total Regals per party-hour in core range with:				
Percent counts with any Regal reported that year in core range	+0.60561	0.0017	+0.60578	<0.01

## Appendix

### Literature Citations of 1990–2014 Fourth of July Butterfly Counts

Hathaway M (ed) (1977) [1977 Butterfly Count Report] Wings 4(1): 1–11.

Hathaway M (ed) (1978) 1977 Butterfly Count Report!!! Wings 4(3) and 5(1): 4–10.

Hathaway M (ed) (1978) 1978 Fourth of July Butterfly Count Report. Wings 5(3): 7–11.

Heller I (1980 [82]) 1980 Butterfly Count Results. Atala Supplement volume 8, The Xerces Society.

Heller I (1980 [82]) 1981 Butterfly Count Results. Atala Supplement volume 8, The Xerces Society.

Opler PA, Brown JW (eds) (1988) Butterfly Counts 1987. Supplement to Atala volume 16. The Xerces Society, Portland, Oregon.

Opler PA, Brown JW (eds) (1989) Fourth of July Butterfly Counts 1988 Report. The Xerces Society, Portland, Oregon.

Opler PA, Brown JW (eds) (1990) Fourth of July Butterfly Counts 1989 Report. The Xerces Society, Portland, Oregon.

Opler PA, Brown JW (eds) (1991) Fourth of July Butterfly Counts 1990 Report. The Xerces Society, Portland, Oregon.

Opler PA, Powell JA (eds) (1984) Butterfly Counts 1982 & 1983. The Xerces Society, Portland, Oregon.

Opler PA, Powell JA (eds) (1985) Butterfly Counts 1984. The Xerces Society, Portland, Oregon.

Opler PA, Powell JA (eds) (1986) Butterfly Counts 1985. Supplement to Atala volume 14. The Xerces Society, Portland, Oregon.

Opler PA, Powell JA (eds) (1987) Butterfly Counts 1986. Supplement to Atala volume 15. The Xerces Society, Portland, Oregon.

Opler PA, Swengel AB (eds) (1992) Fourth of July Butterfly Counts 1991 Report. The Xerces Society, Portland, Oregon.

Opler PA, Swengel AB (eds) (1994) NABA-Xerces Fourth of July Butterfly Counts 1993 Report. North American Butterfly Association, Morristown, New Jersey.

Powell JA, Sorenson JT (eds) (1980) 1979 Butterfly Count Results. Supplement to Atala volume 7 (August 1980), The Xerces Society, Berkeley, California.

Pyle, Sally (1975) Report of the Xerces Society 1st Annual Fourth of July Butterfly Count. Atala Supplement volume 3(2): 38–41.

Swengel AB (ed) (2002) 2001 Report NABA Butterfly Counts. North American Butterfly Association, Morristown, New Jersey.

Swengel AB, Opler PA (eds) (1993) Fourth of July Butterfly Counts 1992 Report. The Xerces Society, Portland, Oregon.

Swengel AB, Opler PA (eds) (1995) NABA-Xerces Fourth of July Butterfly Counts 1994 Report. North American Butterfly Association, Morristown, New Jersey.

Swengel AB, Opler PA (eds) (1996) NABA-Xerces Fourth of July Butterfly Counts 1995 Report. North American Butterfly Association, Morristown, New Jersey.

Swengel AB, Opler PA (eds) (1997) NABA Fourth of July Butterfly Counts 1996 Report. North American Butterfly Association, Morristown, New Jersey.

Swengel AB, Opler PA (eds) (1998) NABA Fourth of July Butterfly Counts 1997 Report. North American Butterfly Association, Morristown, New Jersey.

Swengel AB, Opler PA (eds) (1999) NABA Fourth of July Butterfly Counts 1998 Report. North American Butterfly Association, Morristown, New Jersey.

Swengel AB, Opler PA (eds) (2000) NABA Fourth of July Butterfly Counts 1999 Report. North American Butterfly Association, Morristown, New Jersey.

Swengel AB, Opler PA (eds) (2001). 2000 Report NABA Fourth of July Butterfly Counts. North American Butterfly Association, Morristown, New Jersey.

Swengel AB, Swengel SR (eds) (2003) 2002 Report NABA Butterfly Counts. North American Butterfly Association, Morristown, New Jersey.

Swengel AB, Swengel SR (eds) (2004) 2003 Report NABA Butterfly Counts. North American Butterfly Association, Morristown, New Jersey.

Swengel AB, Swengel SR (eds) (2005) 2004 Report NABA Butterfly Counts. North American Butterfly Association, Morristown, New Jersey.

Wander S (ed) (2006) 2005 Report NABA Butterfly Counts. North American Butterfly Association, Morristown, New Jersey.

Wander S (ed) (2007) 2006 Report NABA Butterfly Counts. North American Butterfly Association, Morristown, New Jersey.

Wander S (ed) (2008) 2007 Report NABA Butterfly Counts. North American Butterfly Association, Morristown, New Jersey.

Wander S (ed) (2009) 2008 Report NABA Butterfly Counts. North American Butterfly Association, Morristown, New Jersey.

Wander S (ed) (2010) 2009 Report NABA Butterfly Counts. North American Butterfly Association, Morristown, New Jersey.

Wander S (ed) (2011) 2010 Report NABA Butterfly Counts. North American Butterfly Association, Morristown, New Jersey.

Wander S (ed) (2012) 2011 Report NABA Butterfly Counts. North American Butterfly Association, Morristown, New Jersey.

Wander S (ed) (2013) 2012 Report NABA Butterfly Counts. North American Butterfly Association, Morristown, New Jersey.

Wander S (ed) (2014) 2013 Report NABA Butterfly Counts. North American Butterfly Association, Morristown, NJ.

Wander S (ed) (2015) 2014 Report NABA Butterfly Counts. North American Butterfly Association, Morristown, NJ.
